# Spike trains in posterior parietal and premotor cortex encode trained and natural grasping behaviors

**DOI:** 10.1186/1471-2202-12-S1-P36

**Published:** 2011-07-18

**Authors:** Esther P Gardner, David Putrino, Jessie Chen

**Affiliations:** 1 Department of Physiology and Neuroscience, New York University School of Medicine, New York, NY 10016, USA

## 

To investigate the role of somatosensory and motor information during grasping behaviors, we used digital video and burst analysis of simultaneously recorded spike trains to define burst epochs when neuronal firing rates exceeded 1 SD above the mean. We reconstructed the trajectory of hand movements during each burst from successive digital video images as three macaques grasped and manipulated objects in a trained prehension task, and when engaged in natural grasping behaviors to acquire pieces of fruit. In the task, neurons in posterior parietal areas 5 and 7b/AIP and in ventral premotor cortex responded more vigorously during object acquisition than to manipulation. Firing rates rose 250-500 ms before touch, and peaked as the hand was preshaped during reach, or at initial contact with the object. Firing rates declined as grasp was secured, and returned to baseline or were inhibited during subsequent actions. Some neurons responded to grasping actions of the right and left hands (bilateral neurons), suggesting that their firing patterns reflect grasp intentions, or the internal motor commands for execution of these behaviors.

**Figure 1:**
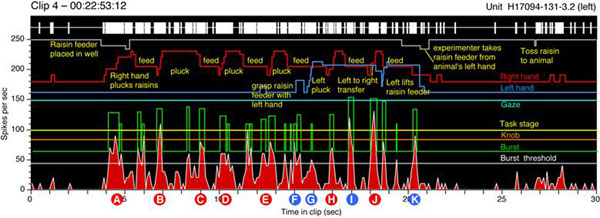


Acquisition-sensitive firing patterns were also observed when the animal grasped food morsels at various workspace locations. Firing began as the animal projected the hand towards the food, and continued as the hand tracked it. Figure 1.

Firing peaked as the fingertips contacted the food, and ended when it was secured in the hand. High firing was elicited when food morsels were plucked from a tray, with the fingers preshaped for precision grip, or during tracking actions when the fingers were spread apart to maximize surface area. As in the task, bilateral neurons responded to prehensile actions performed unilaterally by either hand. A second, weaker burst often occurred when food was placed in the mouth. Other neurons responded vigorously to acquisition by the contralateral hand, but fired at highest rates when bilateral actions were coordinated between the left and right hands, as when food morsels were transferred between them. These intrapersonal-coordinated neurons did not just encode equivalent tactile information from either side, but preferentially signaled coincident somatosensory data shared between hemispheres during synergistic hand actions. The two classes of bilateral neurons thus provide somesthetic feedback from both limbs, and encode whether they are acting independently or in concert.

Our findings support hypotheses that firing patterns in posterior parietal and premotor cortex reflect the animal’s intentions to accomplish task goals in motor coordinates. They suggest that actions preceding contact reinforce subsequent neural responses, allowing subjects to acquire and manipulate objects in a continuous, smooth sequence.

